# Anti-LGI1 encephalitis and co-existence of MOG-IgG: a case report and literature review

**DOI:** 10.3389/fnhum.2025.1585730

**Published:** 2025-07-09

**Authors:** Xiaojiao Ci, Liuyu Lin, Yuqing Wu, Yifang Ma, Jie Lu

**Affiliations:** Department of Neurology, The Affiliated Brain Hospital of Nanjing Medical University, Nanjing, China

**Keywords:** myelin oligodendrocyte glycoprotein antibody, anti-LGI1 antibody, encephalitis, anti-LGI1 and anti-MOG, co-existing antibodies and autoimmune encephalitis, multiple antibodies and autoimmune encephalitis

## Abstract

**Background:**

Anti-leucine-rich glioma-inactivated-1 (LGI1) encephalitis is an autoimmune disorder characterized by antibodies that target LGI1 (LGI1-IgG). It typically presents with cognitive impairment, psychiatric disturbances, and faciobrachial dystonic seizures (FBDS). Myelin oligodendrocyte glycoprotein antibody-associated disease (MOGAD) is currently recognized as a demyelinating disease of the central nervous system (CNS) mediated by antibodies against myelin oligodendrocyte glycoprotein (MOG-IgG). The co-occurrence of anti-LGI1 encephalitis and MOG-IgG is a rare phenomenon.

**Methods:**

We report a case of anti-LGI1 antibody encephalitis combined with MOG-IgG. A comprehensive literature search was conducted using the PubMed and Embase databases. We utilized the following search terms: (“Limbic Encephalitis”[MeSH Terms] OR (“autoimmune encephalitis”[Title/Abstract] OR “AE”[Title/Abstract])) AND (“Myelin-Oligodendrocyte Glycoprotein”[MeSH Terms] OR “demyelinating autoimmune diseases, cns”[MeSH Terms] OR (“MOG-IgG”[Title/Abstract] OR “MOGAD”[Title/Abstract])). The search was constrained to the period from January 1, 2010, to December 31, 2024.

**Results:**

A total of nine papers involving 11 patients were included in the study. Three patients exhibited MOG-IgG in combination with LGI1-IgG. The majority of cases presented with encephalopathic symptoms. Visual changes were observed in a few cases with low titers of serum MOG-IgG or solely in the presence of MOG-IgG in the cerebrospinal fluid (CSF).

**Conclusion:**

The occurrence of anti-LGI1 encephalitis alongside MOG-IgG is a relatively rare phenomenon. The clinical manifestation of encephalopathy in patients with coexisting antibodies presents a significant challenge for clinicians regarding timely diagnosis, highlighting the need for increased vigilance in daily practice.

## Introduction

Anti-leucine-rich glioma-inactivated-1 (LGI1) encephalitis is a synaptic autoimmune disorder mediated by LGI1-IgG. It is considered the second most prevalent autoimmune encephalitis (AE) after anti-N-methyl-D-aspartate receptor encephalitis (NMDARe) (Broadley et al., 2019; Hébert et al., 2020; Shan et al., 2021). Patients with LGI1-IgG often present with acute behavioral changes, psychosis, seizures, faciobrachial dystonic seizures (FBDS), and cognitive impairment (Wang et al., 2018). Myelin oligodendrocyte glycoprotein (MOG) is a protein expressed in the myelin sheath of the central nervous system (CNS). Antibodies against MOG (MOG-IgG) have been identified in acquired CNS demyelinating syndromes with varying clinical manifestations, serving as a crucial diagnostic biomarker for MOG antibody-associated disease (MOGAD). Several studies have reported the coexistence of NMDAR-IgG and MOG-IgG, which has been characterized as MOGAD and NMDARe overlapping syndrome (MNOS) (Fan et al., 2018; Ren et al., 2019; Rojc et al., 2019; Ma and Jiang, 2020; Pérez et al., 2020; Kunchok et al., 2021; Orozco et al., 2023). The presence of NMDAR in oligodendrocytes and the exposure to oncological markers in teratomas provide further support for the existence of MNOS (Bartakova et al., 2016; Jarius et al., 2016a; Zhou et al., 2017). It is important to note that the predominant subtype of the LGI1-IgG is of the IgG4 type. The limited effect of complement and epidemiological data indicating that 5–10% of combined tumors support the rare occurrence of both LGI1-IgG and MOG-IgG (Irani et al., 2012; Huijbers et al., 2015; van Sonderen et al., 2016). Here, we report a case of anti-LGI1 encephalitis combined with positive MOG-IgG. Additionally, a comprehensive review of the literature on autoimmune encephalitis with MOG-IgG coexistence, excluding NMDARe, was conducted.

## Case description

A previously healthy 50-year-old man was admitted to our hospital in December 2021 due to worsening seizures and progressive cognitive impairment. The patient initially presented with a generalized tonic–clonic seizure during sleep in August 2021, without identifiable triggers. He was subsequently taken to a local hospital for a brain magnetic resonance imaging (MRI) scan and a routine electroencephalogram (EEG). Since the results were normal, he chose to be discharged. Ten days later, he experienced another seizure and was prescribed levetiracetam; however, there was no significant improvement in his symptoms. He continued to have monthly convulsions and exhibited progressive memory deterioration, often experiencing lapses and confusion in familiar surroundings. Given the worsening symptoms and lack of improvement, the patient was referred to the neurology clinic at our hospital for further evaluation and management.

At the outpatient clinic, the patient underwent a neurological physical examination, which revealed disorientation concerning time and place. The results of the Mini-Mental State Examination (MMSE) and the Montreal Cognitive Assessment -Basic (MoCA-B) were 22 [normal range: ≥22] and 19 [normal range: ≥22], respectively. The assessment of cranial nerve function, motor examination, and cerebella functions showed no significant abnormalities.

**Figure 1:**
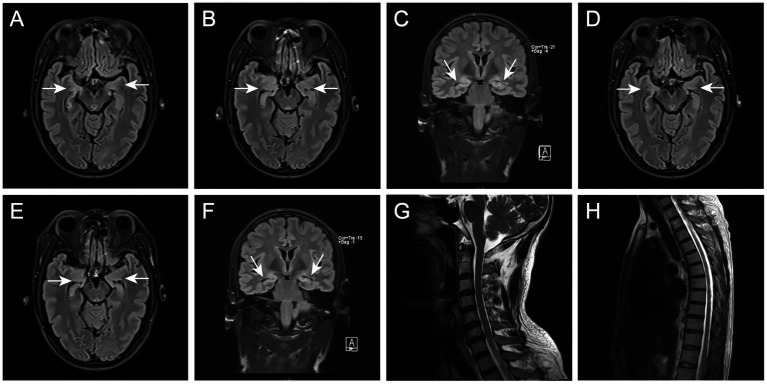
**(A–C)** Brain MRI in December 2021 showed T2-hyperintense lesions at bilateral hippocampus and slight atrophy in right hippocampus. **(D–F)** Brain MRI in April 2022 showed significant hippocampal atrophy. **(G,H)** Spinal cord MRI in April 2022 showed normal.

**Figure 2:**
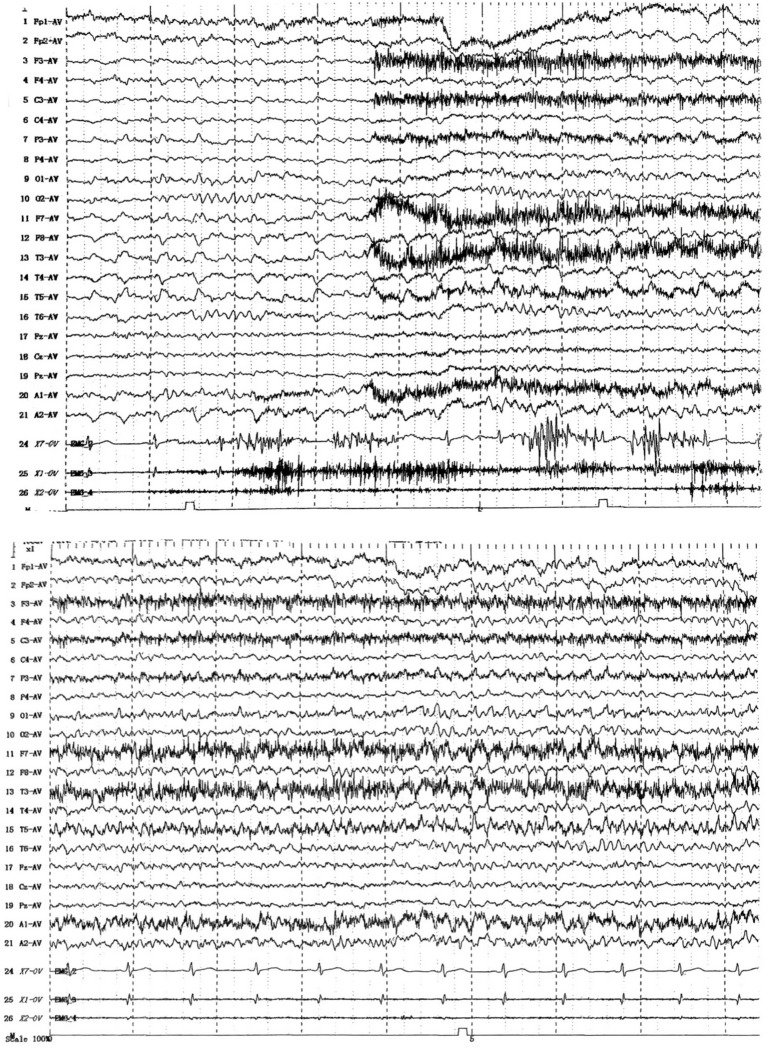
EEG (2021.12).

To determine the underlying causes of the patient’s condition, a comprehensive series of routine clinical biochemical tests was performed, including a complete blood count, erythrocyte sedimentation rate, liver and kidney function tests, as well as systemic autoantibodies assessments. All results returned negative. Brain MRI revealed abnormal hyperintensities bilaterally in the hippocampus. The medial temporal lobe atrophy (MTA) (Scheltens et al., 1992) score was 1, indicating slight atrophy of the hippocampus (Figures 1A–C). There was no evidence of thymoma or other tumors. The video EEG indicated the presence of spike–wave activity in the left temporal region (Figure 2). Examination of the cerebrospinal fluid (CSF) showed a normal opening pressure, with normal leukocyte levels (4 cells/μL), mildly elevated protein levels (0.88 g/L) [normal range: 0.2–0.4], and a normal glucose level (3.72 mmol/L) [normal range: 2.5–4.4]. The results of the immunological analyses, conducted using fixed cell-based assays (Fixed-CBA), indicated the presence of specific LGI1-IgG in both the CSF (1:100) and serum (1:100). No antibodies against NMDAR, AMPAR1, AMPAR2, GABAB, GABAA, CASPR2, IgLON5, DPPX, GlyR1, mGluR5, GAD65, AQP4, MOG, GFAP, Yo, Hu, Ri, Ma2, or amphiphysin were detected in either serum or CSF.

These findings led to the diagnosis of anti-LGI1 encephalitis, followed by treatment with a course of high-dose methylprednisolone and intravenous immunoglobulin. After a one-month hospitalization, the patient was reassessed, achieving scores of 27 on the MMSE and 22 on the MoCA-B. The patient was discharged from the hospital and continued to take low-dose oral glucocorticoids.

Three months later, the patient presented again with hyperhidrosis. Blood tests, liver and kidney function assessments, systemic autoantibodies screenings, and EEG results were consistent with those obtained prior to the initial discharge, revealing no significant abnormalities. A brain MRI indicated hippocampal sclerosis (Figures 1D–F). Re-analysis of the CSF showed a total protein concentration of 1.19 g/L [normal range: 0.2–0.4]and a glucose level of 2.98 mmol/L [normal range: 2.5–4.4], indicating an elevation in the patient’s CSF proteins compared to previous measurements. In light of the emergence of new symptoms, the possibility of a relapse of encephalitis was considered. Serum and CSF samples were subjected to further testing for antibodies against NMDAR, AMPAR1, AMPAR2, GABAB, GABAA, CASPR2, IgLON5, DPPX, GlyR1, mGluR5, GAD65, AQP4, MOG, and GFAP. The results demonstrated LGI1-IgG titers of 1:32 in serum and 1:3.2 in CSF (lower than during the initial presentation). Notably, a new antibody, MOG-IgG, was identified in both serum and CSF (serum titer 1:32, dilution titer 1:10; CSF titer 1:32, dilution titer 1:1). The presence of these novel antibodies in both serum and CSF led to the hypothesis that the patient may have a comorbidity with MOGAD. To further validate this hypothesis, MRI scans of the entire spinal cord were performed; however, no significant abnormal signals were detected on the T2 Flair (Fluid-Attenuated Inversion Recovery) sequence (Figures 1G, H). Given the patient’s lack of other typical symptoms of MOGAD, such as optic neuritis, myelitis, acute disseminated encephalomyelitis (ADEM), cerebral monofocal or polyfocal deficits, brainstem or cerebellar deficits, and cerebral cortical encephalitis often accompanied by seizures, we concluded that the final diagnosis was a relapse of anti-LGI1 encephalitis. High-dose methylprednisolone and intravenous gamma-globulin therapy continued to be administered. Six months after discharge from the hospital, serum antibody titers were re-evaluated and showed the presence of MOG-IgG again (same titer) using the same methodology. The patient continued treatment with low-dose prednisone (20 mg) daily. Eighteen months after the onset of symptoms, the patient did not experience seizures, cognitive impairment, or excessive sweating and tested negative for both anti-LGI1 and anti-MOG antibodies.

### Literature review

A comprehensive literature search was conducted using the PubMed and Embase databases. The following combinations of search terms were employed: (“Limbic Encephalitis”[MeSH Terms] OR (“autoimmune encephalitis”[Title/Abstract] OR “AE”[Title/Abstract])) AND (“Myelin-Oligodendrocyte Glycoprotein”[MeSH Terms] OR “demyelinating autoimmune diseases, cns”[MeSH Terms] OR (“MOG-IgG”[Title/Abstract] OR “MOGAD”[Title/Abstract])) AND 2010/01/01:2024/12/31[Date - Publication].

### Review data

**Figure 3:**
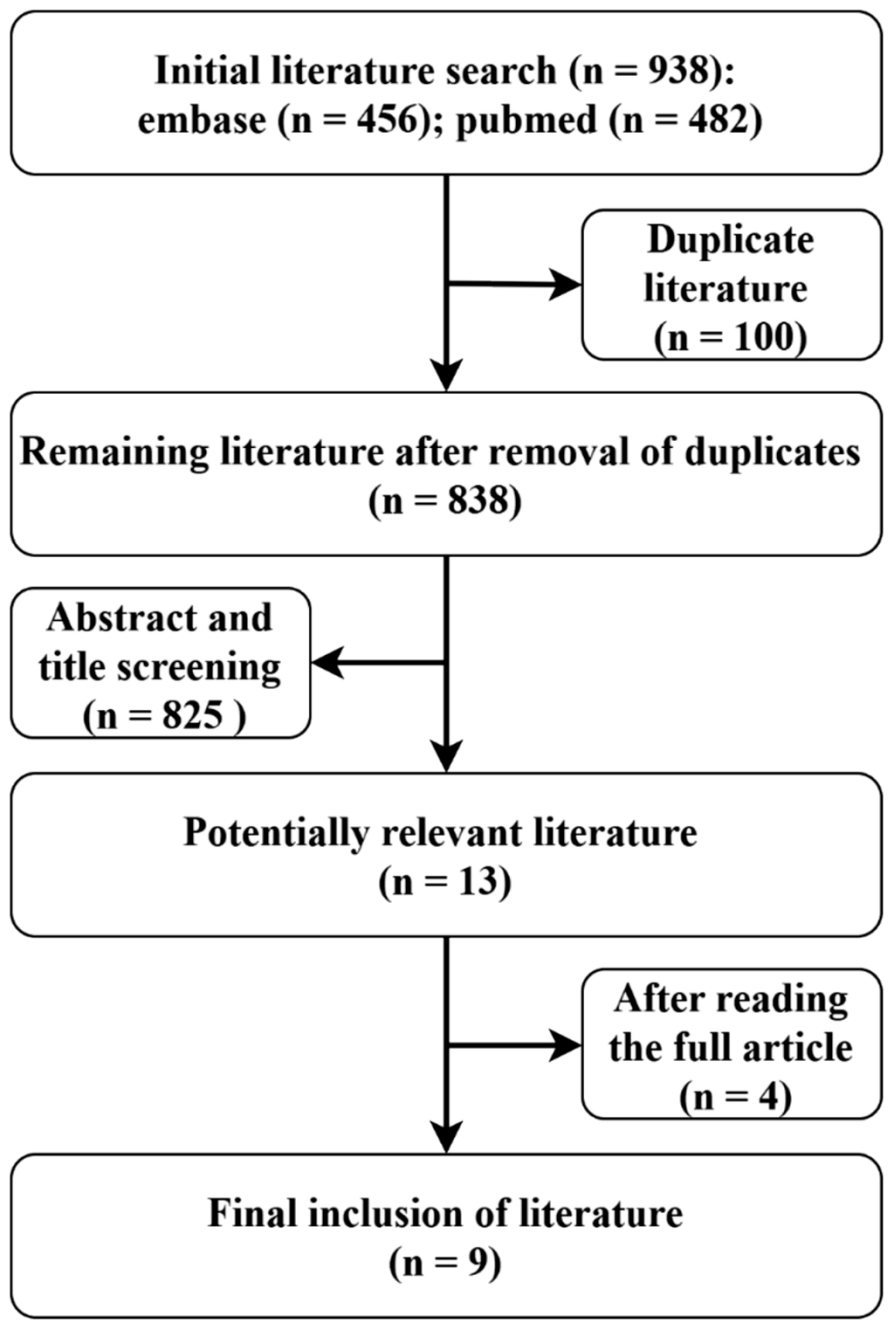
Review flowchart.

A total of 934 studies were identified during the course of this review. Following a comprehensive screening process that included titles, abstracts, and full texts, nine studies (see Figure 3) involving 11 patients were selected for inclusion in this systematic review (Table 1). However, it was noted that two of these patients lacked relevant clinical data. Among the remaining nine patients, two tested positive for anti-CASPR2 antibodies, two for LGI1 antibodies, one for anti-GABAA receptor antibodies, two for GluR antibodies, one for anti-Yo antibodies, and one for anti-IgLON5 antibodies. The collective cases exhibited signs of encephalopathy. Clear MOG-IgG titers were observed in 7 out of 9 patients (1 in CSF and 6 in serum); 3 of these patients demonstrated serum MOG-IgG titers ≥1:100. In Cases 6 and 10, patients presented with headache symptoms. This symptom has been observed in a variety of diseases and is not specific. Two cases exhibited both MOG-IgG and LGI1-IgG: one female and one male, aged 55 and 57 years, respectively. One patient had no documented relevant clinical symptoms, while the other displayed the typical clinical presentation of anti-LGI1 encephalitis, with a baseline MOG-IgG titer of 1:100. However, no distinct MOGAD imaging features were observed, a finding that is consistent with the characteristics of the present case.

**Table 1:** Summary of coexisting MOG-IgG and other antibodies cases include in this study.

Case	Author/years	Sex	Years	Clinical symptoms	Antibodies	Antibodies titers
1	Liu Pei/2020	Female	48	Acute decreased vision, dizziness, slurred speech, gait instability, and urinary incontinence, neuropsychiatric disturbance	MOG, CASPR2	MOG_CSF_:1:1; Caspr2_CSF_:1:1 Caspr2_serum_:1:100
2	Amy Kunchok/2021	Male	10	Ascending paralysis and intractable seizures	MOG, Caspr2	MOG_serum_:1:1000 Caspr2:unknow
3	Amy Kunchok/2021	Female	55	–	MOG, LGI1	MOG_serum_:1:100 LGI1:unknow
4	Amy Kunchok/2021	Male	59	Focal seizures, encephalopathy	MOG, GABAA	MOG_serum_:1:20 GABAA:unknow
5	Amy Kunchok/2022	–	–	–	MOG, LGII	–
6	Yasuhisa Sakurai/2022	Male	45	Abnormal behavior, headache, aphasia, swallowing apraxia, memory impairment, hallucination, generalized tonic convulsion, limb-kinetic apraxia, dysarthria	MOG, GluR	Unknow
7	Dong Xiaojiao/2022	Male	57	Temporal lobe epilepsy, facial arm dystonia, autonomic nerve dysfunction	MOG, LGI1	MOG_serum_:1:100 LGI1_serum_:1:30 LGI1_CSF_: 1:30
8	Li Wanyao/2022	Male	33	Paroxysmal loss of consciousness, significant hypomnesis, difficulty falling asleep, involuntary and movements of the limbs after sleep	MOG, IgLON5	IgLON5Serum:1:30 IgLON5_CSF_:1:10 MOG erum:1:32
9	Ren Manli/2023	Male	58	Unilateral involuntary limb movement	MOG, Yo	YO_serum_:++ MOG_serum_:+
10	He Jianghang/2024	Female	60	Progressive vision loss, headache	MOG, mGluR5	MOG_serum_:1:10 mGluR5_serum_:1:32
11	Yang Jiaxin/2024	–	–	–	MOG, GABAB	–

## Discussion and conclusion

The emergence of antibodies against CNS antigens (e.g., MOG, NMDAR, LGI1, and Caspr2) has not only facilitated the identification of novel disease entities but also expanded our understanding of the underlying mechanisms of CNS autoimmune diseases (Patterson et al., 2018; Ding et al., 2020). The detection of multiple antibodies in individual patients underscores the complexity of these disorders and suggests potential interactions between different autoimmune mechanisms (Jarius et al., 2016b; Cherian et al., 2020; Asioli et al., 2022). In this study, we report a case of anti-LGI1 encephalitis characterized by the development of MOG-IgG positivity during the recovery phase. This atypical manifestation prompts critical inquiries into the underlying pathophysiology, clinical implications, and potential management approaches for similar cases.

### The clinical significance of MOG-IgG in encephalitis

MOGAD is a central nervous system demyelinating disease characterized primarily by optic neuritis, myelitis, and acute disseminated encephalomyelitis (ADEM) (Jarius et al., 2018; Ambrosius et al., 2020; Papathanasiou et al., 2020). Unlike other demyelinating diseases such as multiple sclerosis (MS) or neuromyelitis optica spectrum disorder (NMOSD), MOGAD is primarily influenced by MOG-IgG (Filippi et al., 2024; Uzawa et al., 2024). The clinical manifestations of MOGAD exhibit significant heterogeneity; some patients present with a monophasic course, while others may experience recurrent episodes.

Recognition of MOG-IgG as a biomarker has increased in recent years with the widespread use of CBA. However, the coexistence of MOG-IgG with other autoimmune antibodies (e.g., LGI1-IgG) remains a rare and not fully defined phenomenon. In the case we report, a positive MOG-IgG result in serum and CSF was detected during the recovery phase of anti-LGI1 encephalitis, despite the absence of typical MOGAD symptoms or imaging manifestations. This situation highlights the complexity and challenge of interpreting positive MOG-IgG results in the context of overlapping autoimmune syndromes.

The study by Nguyen et al. (2024) pointed out that in adults, the likelihood of false-positive results significantly increases when the MOG-IgG titer is below 1:40. Other studies have shown that especially in the absence of clinical or imaging evidence of MOGAD, low-titer MOG-IgG results are more likely to be false positives (Jarius et al., 2016a, 2018). This indirectly proves that the clinical significance of a positive MOG-IgG result is usually closely related to the antibody titer and the presence of typical symptoms. In the case we presented, the patient’s MOG-IgG titer was relatively low (serum 1:32, CSF 1:32), and there was a lack of typical MOGAD symptoms, which made us suspect whether it was a false positive result. However, after 6 months of follow-up, the antibodies are still present, which cannot be explained by a false positive. We also conducted a review of other autoimmune encephalitis cases associated with MOG-IgG and found that most patients presented with encephalopathy, with only 2 cases showing signs of optic neuritis. This suggests that in the case of coexisting antibodies, MOG-IgG may not be the primary driver of clinical manifestations. On the contrary, it may represent a secondary or non-specific immune response. This situation highlights the necessity for the establishment of precise antibody titer thresholds within clinical practice, particularly in the context of multiple antibody overlap. Future research endeavors should seek to ascertain titer thresholds for various antibodies through large-scale cohort studies and the development of more precise assays, with the objective of mitigating the impact of false positives on clinical decision-making.

### Why does MOG-IgG appear during the recovery phase of anti-LGI1 encephalitis?—Potential mechanisms

The pathogenic mechanisms underlying the co-existence of LGI1-IgG and MOG-IgG remain speculative. LGI1-IgG are predominantly of the IgG4 subclass (Irani et al., 2012; Huijbers et al., 2015; Crisp et al., 2016), which is generally considered “benign” due to its limited capacity for antibody-dependent cell-mediated cytotoxicity (ADCC), antibody-dependent cellular phagocytosis (ADCP), and complement-dependent cytotoxicity (CDC) (Dalakas, 2022; Rispens and Huijbers, 2023). However, recent studies have shown that IgG4 can activate the complement system via alternative or lectin pathways, leading to the production of inflammatory mediators such as C5a and C3a (Kridin and Ahmed, 2019; Brglez et al., 2020). These fragments can upregulate cytokine levels, potentially exacerbating immune-mediated inflammation.

Animal experiments have indicated that interleukin 3 (IL-3) may be a significant contributor to autoimmune encephalomyelitis, exacerbating autoimmune brain symptoms and intensifying inflammation (Renner et al., 2016). A comparative analysis of cytokine profiles in neuroantibody-mediated CNS disorders revealed that IL-17 and IL-23 were significantly elevated in the serum of patients with autoimmune encephalitis (predominantly anticellular surface antigen) (Ulusoy et al., 2012). They also found Th17-type immunity is another aspect that is more closely related with cell-surface-antibody-associated encephalitis. Similarly, cytokine profiling in MOGAD patients has revealed upregulation of Th1, Th17, and regulatory T cells (Tregs)-associated cytokines (Kaneko et al., 2018). The co-existence of LGI1-IgG and MOG-IgG may thus reflect a complex interplay between these immune pathways, with potential synergistic effects on disease pathogenesis. The emergence of MOG-IgG during the recovery phase of anti-LGI1 encephalitis may suggest ongoing immune activation. This immune response could be driven by the initial autoimmune process or triggered by other factors such as infections or inflammatory mediators. Additionally, studies have shown that 90% of patients with anti-LGI1 encephalitis carry the class II allele HLA-DRB1*07:01, although its role in influencing the immune response remains unclear (Kim et al., 2016; Van Sonderen et al., 2017; Binks et al., 2018). Future research should focus on exploring the interactions between LGI1-IgG and MOG-IgG at the molecular and cellular levels, as well as the role of cytokines and complement activation in patients with dual antibody positivity. Understanding these mechanisms could provide valuable insights into the pathogenesis of overlapping autoimmune syndromes and inform the development of targeted therapeutic strategies.

### Conclusion

In conclusion, we present a rare case of anti-LGI1 encephalitis with the emergence of MOG-IgG positivity during the recovery phase. This unusual presentation highlights the complexity of autoimmune encephalitis and the potential for overlapping antibody profiles. The possibility of MOG-IgG false positivity cannot be entirely excluded, especially given the low antibody titers and atypical clinical presentation. Clinicians should remain vigilant for the co-occurrence of multiple antibodies in patients with autoimmune encephalitis and consider the possibility of false positives in low-titer settings. Further research is needed to elucidate the underlying mechanisms and optimize management strategies for these challenging cases.

## Data Availability

The original contributions presented in the study are included in the article/supplementary material, further inquiries can be directed to the corresponding author.
